# East Texas Medico-Chirurgical Society

**Published:** 1900-06

**Authors:** 


### East Texas Medico-Chirurgical Society.

The East Texas Medico-Chirurgical Society will meet in Palestine, Texas, July 5, prox., at 2 p. m.

Chairman of Program Committee—Dr. E. M. Link, Palestine.

PROGRAM.

Invocation by Rev. Dr. R. H. Crozier, Palestine.
Address of Welcome by Hon. A. W. Gregg, Palestine.
Response by Dr. F. B. Moore, Brushy Creek.
Roll call.
Reading minutes of last meeting by Secretary.
Report of Treasurer Dr. John Colly.
Applications for membership.
Report of Board of Censors.
Adjournment until 8 p. m.

FIRST DAY—EVENING SESSION.

*Section on General Medicine:*

Dr. E. P. Murdock, Oakwood, Texas—"Typhoid Fever and its Treatment."

Dr. J. N. Gee, Bethal, Texas—"Diphtheria."

Refreshments.

SECOND DAY—MORNING SESSION.

Dr. J. H. Joyce, Buffalo—"General Medicine."

Dr. W. T. Evans, Jewett, Texas—"Scarlet Fever, or Something Similar."

*Section on Surgery:*

Dr. J. H. McDaniel, Centerville—"Gun Shot Wounds of Abdomen and Their Treatment."

Dr. W. G. Jameson, Palestine—"Fractures and Dislocations of Elbow and Their Treatment."

SECOND DAY—AFTERNOON SESSION.

Dr. E. E. Guinn, Rusk—"Treatment of Hernia."

Dr. W. C. Lipscomb, Crockett—"Aseptic vs. Septic Surgery."

*Section on Gynecology and Obstetrics:*

Dr. A. R. Link, Palestine—"Cause and Treatment of Uterine Displacements."

Dr. L. Merriweather, Grapeland—"Post-Partum Hemorrhage."
Dr. F. B. Moore, Brushy Creek—"Cause and Treatment of Puerperal Sepsis."
Dr. E. B. Stokes, Crockett—"Metritis and its Treatment."
General business.
Adjournment.

SAM R. BURROUGHS, President.

E. E. GUINN, Secretary.

DR. E. M. LINK,
DR. A. L. HATHCOCK,
DR. J. H. JONES,
Committee on Program.

